# A longitudinal study of symptom cluster latent profiles in ovarian cancer patients undergoing chemotherapy

**DOI:** 10.1002/cam4.7139

**Published:** 2024-03-28

**Authors:** Yue Feng, Tangwei Lin, Xingcan Liu, Xiujing Guo, Jing Chen, Xue Deng

**Affiliations:** ^1^ Department of Gynecological Nursing, West China Second University Hospital Sichuan University Chengdu China; ^2^ Key Laboratory of Birth Defects and Related Diseases of Women and Children, Ministry of Education Sichuan University Chengdu China

**Keywords:** fatigue, pain, sleep–wake disorders, symptom cluster

## Abstract

**Background:**

This study aimed to identify distinct patterns within the symptom cluster of fatigue, pain, and sleep disturbance among ovarian cancer patients receiving chemotherapy, to determine the factors predicting these patterns and their impact on quality of life.

**Method**s**:**

The longitudinal study collected data from 151 ovarian cancer patients at three time points: before chemotherapy (T0), after the first chemotherapy cycle (T1), and following the completion of four cycles of chemotherapy (T2). Latent profile analysis and latent transition analysis were used to identify symptom patterns and evaluate changes in symptom patterns. A bias‐adjusted three‐step approach was utilized to examine predictor variables and distal outcomes associated with latent class membership.

**Results:**

Three symptom patterns emerged: “All Low,” “Moderate” (T0)/“Low pain and high sleep disturbance” (T1 and T2), and “All High.” Patients with lower educational attainment and higher levels of anxiety and depression were found to be at an elevated risk of belonging to the “All High” class. All quality‐of‐life domains showed significant differences among the three subgroups, following an “All Low” > “All High” pattern (*p* < 0.05). Membership in three classes remained relatively stable over time, with probabilities of 0.749 staying within their groups from T0 to T2.

**Conclusions:**

This study underscores the existence of a diverse and heterogeneous experience within the symptom cluster of fatigue, pain, and sleep disturbance among ovarian cancer patients. Importantly, these patterns were stable throughout chemotherapy. Recognizing and understanding these patterns can inform the development of targeted interventions to alleviate the burden of symptom clusters in this population.

## 1 INTRODUCTION

Ovarian cancer survivors commonly face a range of symptom clusters linked to the disease and its treatments, which can lead to physical and psychological challenges, ultimately affecting their overall quality of life.^1^, ^2^, ^3^ Among these symptom clusters, one of the most prevalent and severe is characterized by “fatigue, pain, and sleep disturbances,” a combination often encountered by cancer patients.^4^, ^5^

Previous studies on symptom clusters in cancer patients predominantly used two conceptual approaches. The first is a “variable‐centered” analytical method, assuming that symptoms were uniformly distributed and grouping symptoms to create symptom clusters.^6^ This approach's most common statistical analyses were principal components analysis^7^ and factor analysis.^8^ However, this approach may not adequately capture the diversity of symptom experiences among cancer patients, as it overlooks individual differences.^9^ Another method, such as latent profile analysis (LPA), focused on “person‐centered” and grouped patients by symptom experiences. LPA is designed to categorize individuals into discrete latent groups based on observable variables derived from continuous indicators. LPA's strength lies in its ability to uncover hidden patterns in the data by maximizing homogeneity within each latent group while highlighting the heterogeneity between these groups.^9^

Up to this point, various studies have contributed valuable insights into various aspects of symptom clusters, particularly in their attempts to categorize patients based on their experiences with the symptom cluster of fatigue, pain, and sleep disturbance. Some of these studies have primarily focused on diverse cancer populations, failing to account for the unique subpopulation differences within the symptom cluster of fatigue, pain, and sleep disturbance.^10^, ^11^, ^12^ Studies have also emerged for specific subpopulations, such as breast, lung, and pediatric cancer,^13^, ^14^, ^15^ that used LPA to classify patients into latent classes based on their unique experiences with some of the common co‐occurring symptoms during chemotherapy.^16^, ^17^ Notably, these studies have identified a subgroup of individuals with high‐symptom burden. This subgroup may represent a high‐risk category with a distinct symptom phenotype.^11^

The prevalence rates for fatigue, pain, and sleep disturbance in ovarian cancer patients during chemotherapy varied widely. Specifically, the prevalence ranged from 69.78% to 85.26% for fatigue, 50.93% to 79.35% for pain, and 67.37% to 74.07% for sleep disturbance.^8^, ^18^ Previous studies of patients with gynecological cancer have used LPA to identify subgroups of single symptoms such as anxiety^19^ and sleep disturbance^20^ during chemotherapy and explored the risk factors of the susceptible to symptom burden. However, few studies assessed the profiles for the occurring symptoms only in patients with ovarian cancer receiving chemotherapy. Therefore, there remains a critical need for similar investigations to provide evidence specific to ovarian cancer patients experiencing the symptom cluster of fatigue, pain, and sleep disturbance.

Moreover, it is worth noting that many studies examining symptom clusters in cancer patients have been designed cross‐sectionally. A longitudinal approach is essential to gain a more comprehensive understanding of the dynamics of latent symptom clusters, particularly in the context of the symptom cluster of fatigue among ovarian cancer patients undergoing chemotherapy. The LPA has been extended to latent transition analysis (LTA), which can simultaneously estimate latent classes and their development trends over time.^21^ The LTA calculates the transition probability of shifting one latent class to another over time and enables evaluations of the likelihood of change in patient symptoms following chemotherapy. We can monitor the consistency of an individual's symptom profile from their initial baseline assessment to subsequent observations, determining whether they maintain the same profile or transition to a different one using LTA.^22^ This approach enables the identification of latent symptom clusters at distinct time points and offers insights into how these latent groups may evolve. Such longitudinal studies hold the potential to inform the development of proactive care strategies tailored to the unique needs of ovarian cancer patients facing the challenges of the fatigue symptom cluster.^13^

Hence, our study was designed as a longitudinal investigation aimed at delineating subgroups within ovarian cancer patients undergoing chemotherapy, explicitly focusing on their experiences and change patterns with fatigue, pain, and sleep disturbance. The other two objectives were to find potential factors associated with membership in these latent subgroups and to determine whether the presence of different latent classes was linked to distinct quality of life among these patients. This approach allowed us to gain a more comprehensive understanding of the symptom experiences of ovarian cancer patients and their subsequent impact on overall well‐being.

## 2 METHODS

### 2.1 Participants and procedures

We recruited participants from March 2021 to May 2022 at a women's and children's hospital. Patients were enrolled if they (1) had a confirmed diagnosis of ovarian cancer; (2) had completed total staging surgery, cytoreductive tumor surgery, or maximal resection and were scheduled to begin their first round of chemotherapy; and (3) could understand and answer relevant questions. Patients were excluded if they (1) had severe psychiatric disease, (2) had more than one cancer diagnosis, and (3) had a sleep disorder diagnosis.

Data were collected at three specific time points during chemotherapy: enrollment before the commencement of chemotherapy (T0) and 1 week after the first and the fourth cycle of chemotherapy (T1 and T2). Patients were contacted by telephone or the WeChat platform to complete the questionnaires.

### 2.2 Sample size

Previous studies^23^, ^24^ indicated that the aBIC (adjusted Bayesian information criteria) was the most accurate indicator of the information criteria. Precisely, aBIC needed at least 50 subjects per latent class to be accurate (>90%) in the simulation study. The prior research focused on fatigue, pain, and sleep disturbance using latent profile analysis, often dividing the symptom cluster into three subgroups. Therefore, our study aimed to include at least 150 subjects to ensure our investigation's statistical power and reliability. Considering a conservative dropout rate of approximately 20%, 188 participants were proposed to be recruited for our study.

### 2.3 Measures

#### 2.3.1 Symptoms assessment

The Functional Assessment of Chronic Illness Therapy‐Fatigue (FACIT‐F) scale was used to evaluate fatigue. The FACIT‐F comprises 13 items that assess patients' self‐reported fatigue during their daily activities over the past 7 days.^25^ The FACIT‐F was scored on a 5‐point Likert scale ranging from 0 (not at all) to 4 (very much), with Items 7 and 8 being reverse‐scored. The scale has a total score of 52, with lower scores reflecting more severe fatigue.^26^ The FACIT‐F has been validated in cancer patients with a reliability of 0.84–0.90 and an internal consistency of 0.93–0.95.^27^

Pain intensity was assessed using a 0 (no pain) to 10 (worst pain imaginable) numeric rating scale (NRS). NRS is a valid and reliable measure of pain intensity.^28^

We used the Pittsburgh Sleep Quality Index (PSQI) to assess sleep disturbances. It is a validated self‐report instrument consisting of 19 items in seven clinical domains of sleep difficulty: subjective sleep quality, sleep latency, sleep duration, sleep efficiency, sleep disturbances, use of sleep medications, and daytime dysfunction.^29^, ^30^ The total scores were calculated by adding the scores for each domain (ranging from 0 to 21), with higher scores reflecting more severe sleep disturbances. The PSQI has a Cronbach's *α* of 0.83, with high sensitivity (99%) and specificity (84%) to identify sleep disturbances.^30^

#### 2.3.2 Anxiety and depression

Anxiety and depression levels were measured using the Hospital Anxiety and Depression Scale (HADS). The HADS is a widely used scale to assess anxiety and depression (seven items for each construct) in inpatients and has been tested for validity and accuracy.^31^ Items were scored on a 4‐point scale, ranging from 0 to 3. A higher score indicates a higher level of anxiety and depression.

#### 2.3.3 Quality of life

Quality of life was assessed using the European Organization for Research and Treatment of Cancer Quality‐of‐Life‐Questionnaire‐Core‐30 (EORTC‐QLQ‐C30). It includes five functional measures (physical, role, cognitive, emotional, and social), eight symptom measures (fatigue, pain, nausea/vomiting, appetite loss, constipation, diarrhea, insomnia, and dyspnea), one global health/quality of life measure, and financial impact measure. The two items on global health were scored from 1 (very bad) to 7 (very good), and the others were scored from 1 (not at all) to 4 (very much). Raw scores were transformed to a 0–100 scale following recommended guidelines, with higher scores representing better quality of life and greater symptom burden.^32^ The comparison of symptom measures between latent classes was not performed in our study as some of the measures (fatigue, pain, and insomnia) overlapped with our symptom assessment.

#### 2.3.4 Demographic and clinical information

Demographic and clinical information was collected from the medical record of patients' self‐reports, including age, BMI, educational background, marriage status, working status, pathology, and cancer stage, whether there was tumor metastasis and chemotherapy regimen.

### 2.4 Statistical analysis

Latent profile analysis (LPA) and latent transition analysis (LTA) were conducted in Mplus 8.3. First, several LPA models were conducted at three time points to determine the number of categories of the subsequent LTA model. LPA separates the dependency between indicators into dependencies within and between groups by maximizing homogeneity within the latent classes and heterogeneity between the latent classes. Model‐fit indices were used to decide on the best fitting model,^33^ including Log‐Likelihood, Information Criteria (Akaike information criteria [AIC], Bayesian information criteria [BIC], and sample size‐adjusted Bayesian Information Criteria [aBIC]), entropy values, Likelihood‐ Ratio Tests (Lo–Mendell–Rubin Likelihood‐ratio test [LMR], and Bootstrap Likelihood‐Ratio Test [BLRT]). The selection of preferred models is based on the *p*‐value of the LRT and BLRT criteria. A significant *p*‐value suggests that the goodness of fit of the *k*‐cluster model is better than that of the *k*−1 cluster model. A good model fit was evaluated for (1) lower values of the information criterion, (2) statistically significant values of the LMR and BLRT, (3) feasibility of class interpretation, and (4) entropy values >0.80. However, in practice, there is no consistency between the evaluation metrics. In this case, the best classification result should be selected by combining the classification's practical significance with each indicator's specific effects. Additionally, the number of subjects in each potential category was greater than 5% of the population.^34^ It is important to consider the consistency of the symptom cluster classifications across time since data was collected at three time points.

Descriptive statistics and frequency distributions were calculated using SPSS 26.0. A bias‐adjusted three‐step approach was used to analyze latent class predictor variables (demographic, clinical characters, and anxiety and depression symptoms). As BMI, anxiety, and depression differed at each time point, the variables corresponding to the time points were used. The other variables used were data collected at baseline. The Lanza, Tan, and Bray method was used to analyze latent class distal variables (quality of life). They were implemented in Mplus. These methods can independently evaluate the relationship between latent classes and the predictor or distal auxiliary variables.^35^ All statistical tests were two‐tailed, and statistical significance was set at *p* < 0.05.

Finally, following identifying the most suitable latent profile model at each time point, we extended the latent profile analysis (LPA) models to latent transition analysis (LTA) to investigate the transition probabilities of moving from one profile membership to another between T0 to T1, and between T1 and T2. Model comparison and confirmation were conducted using the log‐likelihood.^36^

## 3 RESULTS

### 3.1 Patient information

We recruited 200 ovarian cancer patients in total, where 154 agreed and provided written consent forms. Regrettably, three participants were lost to follow‐up after the initial chemotherapy session, rendering them ineligible for further analysis. Consequently, our final dataset consisted of 151 dedicated patients who finished the questionnaires at all three time points. Table 1 presents the detailed information of our participants. All of them were ethnic Han Chinese with a mean age of 52.79 ± 12.01, ranging from 18 to 76 years. Most participants were married (83.4%), while only 43% reported current employment status. Most of our participants had epithelial ovarian cancer (95.4%), and 60.3% had Stage III or IV tumors.

**TABLE 1:** Demographic and clinical characteristics of participants (*n* = 151).

Characteristics	
Age (mean ± SD)	52.79 ± 12.01
BMI‐T0 (mean ± SD)	22.05 ± 3.00
BMI‐T1 (mean ± SD)	21.99 ± 2.94
BMI‐T2 (mean ± SD)	22.09 ± 3.43
Education background (%)	
Junior high school and below	62 (41.1%)
Senior high school and upper	89 (58.9%)
Work status (%)	
Retired/unemployed	86 (57.0%)
Employed	65 (43.0%)
Marriage status (% yes)	126 (83.4%)
Pathology (%)	
Epithelial	144 (95.4%)
Non‐epithelial	7 (4.6%)
Stages (%)	
Stage I, II	60 (39.7%)
Stage III, IV	91 (60.3%)
Metastasis (% yes)	41 (27.2%)
Chemotherapy regimen	
TP	141 (93.4%)
Others	10 (6.6%)
Anxiety‐T0 (median [P_25_, P_75_])	2.0 [0, 5.0]
Anxiety‐T1 (median [P_25_, P_75_])	3.0 [1.0, 4.0]
Anxiety‐T2 (median [P_25_, P_75_])	3.0 [1.0, 5.0]
Depression‐T0 (median [P_25_, P_75_])	2.0 [0, 4.0]
Depression‐T1 (median [P_25_, P_75_])	2.0 [1.0, 4.0]
Depression‐T2 (median [P_25_, P_75_])	3.0 [1.0, 4.0]

Abbreviation: TP, paclitaxel combined with platinum‐based drugs.

### 3.2 Latent class solution

LPA models were conducted first to determine the number of categories of the subsequent LTA models. Using LPA, we identified three latent profile classes of our participants based on their experiences with fatigue, pain, and sleep disturbance at each time point during chemotherapy. The fit indices of all candidate models are presented in Table S1. It is important to note that the support for specific models varied among the different model‐fitting indices, leading to some inconsistency. At the initial time point, T0, both the AIC and aBIC indices favored the five‐class solution, while the BIC favored a 4‐class solution. Despite these indications, the *p*‐values associated with the LMR suggested that the four‐class and five‐class solutions did not significantly improve the fit compared to the three‐class model. The *p*‐values of both LMR and BLRT supported that the fit of the three‐class model was significantly enhanced compared to the two‐class model. Furthermore, to ensure that each identified category had a sufficient proportion of participants (more than 5% of the total sample), we selected the three‐class solution for T0. The BIC indices and *p*‐values of both LMR and BLRT supported selecting three classes at T1 and T2. Moreover, the entropy of the three‐class was larger than 0.8 at T2 and approximately close to 0.8 at T1, indicating a good model fit in the classification. Considering the consistency of the symptom cluster classifications across time, three‐class solutions were selected for both T1 and T2. The distribution of demographic, clinical characteristics, and anxiety and depression symptoms between latent classes from T0 to T1 were presented in Table S2.

**FIGURE 1:**
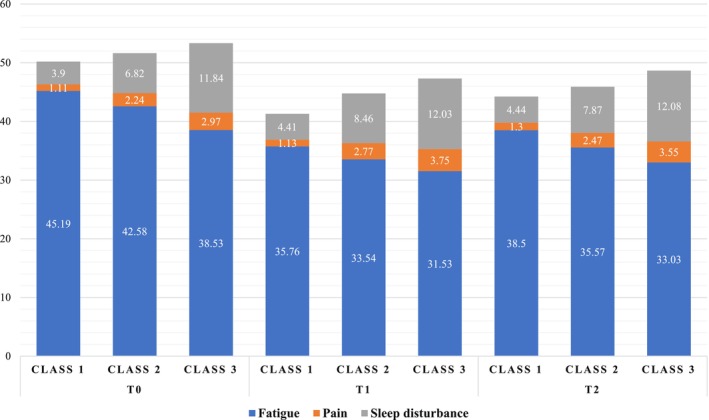
Comparison of scores for each symptom in the three latent classes of ovarian cancer patients during chemotherapy.

The naming of the three classes at each time point was determined based on their respective scores on each symptom measurement scale. Figure 1 presents the average scores for each symptom measurement scale at each time point. Table 2 offers a detailed comparison of symptom severity scores between the latent classes at each time point. Through multiple comparisons, Class 1 consistently exhibited the lowest fatigue, pain, and sleep disturbances across all time points and was named the “All Low” class. Class 3 consistently demonstrated the highest fatigue, pain, and sleep disturbances at all three time points. Hence, it was appropriately labeled the “All High” class. Class 2 displayed a pattern of moderate fatigue, pain, and sleep disturbances at T0, transitioning to low pain and high sleep disturbance at T1 and T2. Consequently, it was named the “Moderate” class at T0 and the “Low Pain and High Sleep Disturbance” class at T1 and T2.

**TABLE 2:** Symptom severity scores compared by latent classes from latent profile analysis (*n* = 151).

		Fatigue		Pain		Sleep disturbance	
	Latent classes	Median [P_25_, P_75_]	Omnibus test *p*‐value; post hoc contrasts	Median [P_25_, P_75_]	Omnibus test *p*‐value; post hoc contrasts	Median [P_25_, P_75_]	Omnibus test *p*‐value; post hoc contrasts
T0	C1 (*n* = 50)	46.0 [43.0, 50.0]	*H* = 19.320** C1 > C2 > C3	0 [0]	*H* = 121.209** C1 < C2 < C3	5.0 [3.0, 6.0]	*H* = 24.482** C1 < C2 < C3
	C2 (*n* = 89)	44.0 [38.0, 48.0]		2.0 [2.0, 3.0]		7.0 [5.0, 10.0]	
	C3 (*n* = 12)	35.0 [23.5, 42.0]		6.0 [6.0, 6.0]		12.0 [7.5, 13.5]	
T1	C1 (*n* = 88)	34.5 [30.0,41.0]	*H* = 10.876* C1 > C3	2.0 [2.0, 2.0]	*H* = 89.683** C1 < C3, C2 < C3	5.0 [4.0, 7.0]	*H* = 100.549** C1 < C2, C1 < C3
	C2 (*n* = 25)	33.0 [30.0, 36.0]		2.0 [2.0, 3.0]		11.0 [10.0, 12.0]	
	C3 (*n* = 38)	31.0 [26.0, 34.0]		5.0 [5.0, 6.0]		11.0 [8.0, 12.0]	
T2	C1 (*n* = 103)	38.0 [32.0, 42.0]	*H* = 17.735** C1 > C3	2.0 [2.0, 2.0]	*H* = 72.388** C1 < C3, C2 < C3	5.0 [4.0, 7.0]	*H* = 80.070** C1 < C2, C1 < C3
	C2 (*n* = 19)	37.0 [32.0, 42.0]		2.0 [2.0, 3.0]		13.0 [11.5, 14.0]	
	C3 (*n* = 29)	32.0 [26.0, 34.0]		5.0 [5.0, 6.0]		10.0 [8.0, 11.0]	

* *p* < 0.05.

** *p* < 0.001.

### 3.3 Predictor variables of latent class membership

Table 3 presents the results of the three‐step approach analyzing predictor variables associated with latent classes at each time point. Considering “All Low” class as a reference, patients who reported severe anxiety experience (OR = 1.323, *p* < 0.05) were more likely to belong to the “Moderate” group. Patients with lower educational attainment (OR = 1/0.332 = 3.012, *p* < 0.05), with elevated levels of anxiety (OR = 1.362, *p* < 0.05), and higher depression scores (OR = 1.250, *p* < 0.05) were at a greater risk of being part of the “All High” class before commencing chemotherapy at T0.

**TABLE 3:** Effect of demographic, clinical characteristics, and anxiety depression symptoms on latent classes using the bias‐adjusted three‐step approach.

Variables	Reference^a^	T0			T1			T2		
		C1^a^ versus C2 OR (95%CI)	C1^a^ versus C3 OR (95%CI)	C2^a^ versus C3 OR (95%CI)	C1^a^ versus C2 OR (95%CI)	C1^a^ versus C3 OR (95%CI)	C2^a^ versus C3 OR (95%CI)	C1^a^ versus C2 OR (95%CI)	C1^a^ versus C3 OR (95%CI)	C2^a^ versus C3 OR (95%CI)
Age	Continuous	0.981 (0.947, 1.016)	0.994 (0.945, 1.046)	1.013 (0.968, 1.060)	1.024 (0.990, 1.059)	1.009 (0.974, 1.045)	0.985 (0.943, 1.028)	1.033 (0.982, 1.087)	1.013 (0.972, 1.056)	0.981 (0.923, 1.042)
BMI	Continuous	0.974 (0.852, 1.113)	1.054 (0.856, 1.297)	1.082 (0.898, 1.303)	0.978 (0.754, 1.269)	1.028 (0.886, 1.193)	1.051 (0.761, 1.452)	0.933 (0.774, 1.124)	0.955 (0.808, 1.128)	1.024 (0.784, 1.337)
Education background	Junior high school and below	1.088 (0.469, 2.522)	0.332 (0.212, 0.520)*	0.306 (0.206, 0.454)*	3.037 (0.027, 337.225)	0.476 (0.315, 0.720)*	0.157 (0.120, 0.205)*	2.611 (0.035, 194.753)	0.476 (0.300, 0.756)*	0.182 (0.129, 0.257)**
Work status	Retired/unemployed	0.865 (0.449, 1.668)	0.581 (0.266, 1.268)	0.672 (0.283, 1.595)	0.800 (0.313, 2.046)	0.913 (0.419, 1.988)	1.142 (0.242, 5.382)	1.189 (0.263, 5.367)	0.479 (0.289, 0.793)*	0.403 (0.210, 0.773)
Marriage status	Married	1.070 (0.358, 3.200)	1.054 (0.171, 6.485)	0.985 (0.197, 4.924)	0.491 (0.199, 1.212)	0.620 (0.296, 1.301)	1.262 (0.085, 18.795)	n/a^b^	0.836 (0.311, 2.249)	n/a^b^
Stages of cancer	Stage I, II	0.494 (0.331, 0.737)	0.577 (0.269, 1.239)	0.857 (0.297, 2.470)	1.085 (0.291, 4.050)	0.533 (0.338, 0.842)	0.491 (0.249, 0.967)	0.560 (0.273, 1.147)	0.459 (0.295, 0.713)	0.820 (0.232, 2.897)
Metastasis	No	0.452 (0.309, 0.661)	0.861 (0.270, 2.747)	1.905 (0.154, 23.597)	0.834 (0.266, 2.615)	1.353 (0.394, 4.651)	1.622 (0.135, 19.471)	0.570 (0.233, 1.393)	0.570 (0.294, 1.106)	1.001 (0.142, 7.037)
Anxiety	Continuous	1.323 (1.027, 1.704)*	1.362 (0.961, 1.931)*	1.030 (0.835, 1.270)	0.722 (0.565, 0.922)	0.765 (0.670, 0.874)	1.384 (0.865, 2.215)	1.235 (0.830, 1.839)	1.382 (1.042, 1.833)*	1.119 (0.739, 1.695)
Depression	Continuous	1.168 (0.960, 1.421)	1.250 (0.978, 1.597)*	1.071 (0.907, 1.265)	1.106 (0.807, 1.516)	1.378 (1.072, 1.771)*	1.246 (0.872, 1.780)	1.37 (0.861, 2.180)	1.427 (0.966, 2.108)*	1.042 (0.760, 1.429)

*Note*: Class 1 = all low class, Class 2 = moderate class at T0, low pain and high sleep disturbance class at T1 and T2, Class 3 = all high class.

^a^ Reference group.

^b^ Odds ratio results cannot calculate and were shown as not applicable.

* *p* < 0.05.

** *p* < 0.001.

After the first cycle of chemotherapy (T1), patients with lower educational levels (OR = 1/0.476 = 2.101, *p* < 0.05) reported experiencing severe depression symptoms (OR = 1.378, *p* < 0.05) were more likely to belong to the “All High” class compared with all low class. After four cycles of chemotherapy (T2), lower educated patients (OR = 1/0.476 = 2.101, *p* < 0.05), retired and unemployed patients (OR = 1/0.479 = 2.088, *p* < 0.05) with severe anxiety (OR = 1.382, *p* < 0.05), and depression symptoms (OR = 1.427, *p* < 0.05) were more likely to be in the “All High” class compared to “All Low” class  .

### 3.4 Quality of life

The results of our quality‐of‐life comparisons, as presented in Table 4, revealed statistically significant differences (*p* < 0.05) in various aspects of quality of life, including physical, role, emotional, cognitive, and social function as well as the overall quality of life among the three latent classes at all three time points. Differences in physical function among the three latent groups followed the “All Low” > “Moderate” > “All High” pattern before chemotherapy. For role function, emotional function, social function, and global health/quality of life scores, differences among the three classes followed “All Low” “>” “Moderate” and “All Low” > “All High.” As for cognitive function scores, differences followed “All Low” > “All High,” “Moderate” “All High.”

**TABLE 4:** Comparison of quality of life by latent class using Lanza, Tan, and Bray approach.

	Domain	Latent Classes	Mean (SE)	Differences between groups^a^			Overall test^a^
				Class 1	Class 2	Class 3	
T0	Physical function	Class 1	89.34 (1.64)	–			36.76**
		Class 2	77.05 (1.86)	24.56**	–		
		Class 3	62.91 (5.91)	18.60**	5.21*	–	
	Role function	Class 1	88.14 (2.19)	–			23.29**
		Class 2	74.05 (2.20)	20.64**	–		
		Class 3	70.74 (6.19)	7.03*	0.26	–	
	Emotional function	Class 1	90.55 (1.56)	–			23.18**
		Class 2	81.24 (1.66)	16.74**	–		
		Class 3	70.38 (6.14)	10.15**	2.92	–	
	Cognitive function	Class 1	88.93 (1.91)	–			7.73*
		Class 2	85.65 (1.62)	1.71	–		
		Class 3	73.51 (5.37)	7.33*	4.70*	–	
	Social function	Class 1	83.88 (2.21)	–			32.74**
		Class 2	67.59 (2.11)	28.43**	–		
		Class 3	62.50 (6.00)	11.20**	0.64	–	
	Global health/quality of life	Class 1	72.29 (2.23)	–			9.29*
		Class 2	64.86 (2.15)	5.76*	–		
		Class 3	54.17 (7.44)	5.44*	1.90	–	
T1	Physical function	Class 1	81.08 (1.27)	–			38.45**
		Class 2	69.83 (3.06)	11.58*	–		
		Class 3	62.86 (2.98)	31.74**	2.67	–	
	Role function	Class 1	75.67 (1.88)	–			21.70**
		Class 2	65.63 (3.97)	5.22*	–		
		Class 3	57.49 (3.65)	19.62**	2.29	–	
	Emotional function	Class 1	85.63 (1.29)	–			17.69**
		Class 2	80.42 (2.43)	3.59	‐		
		Class 3	75.29 (2.17)	16.80**	2.49	–	
	Cognitive function	Class 1	87.93 (1.44)	–			16.15**
		Class 2	76.53 (3.10)	11.12*	–		
		Class 3	79.08 (2.75)	8.12*	0.38		
	Social function	Class 1	74.88 (1.80)	–			15.63**
		Class 2	63.47 (3.59)	8.07*			
		Class 3	62.50 (3.30)	10.87*	0.04		
	Global health/quality of life	Class 1	64.36 (1.28)	–			33.91**
		Class 2	55.10 (2.30)	12.39**	–		
		Class 3	50.74 (2.20)	28.72**	1.87	–	
T2	Physical function	Class 1	81.30 (1.18)	–			37.96**
		Class 2	64.12 (4.54)	13.43**	–		
		Class 3	67.76 (2.24)	28.61**	0.52	–	
	Role function	Class 1	75.04 (1.79)	–			21.42**
		Class 2	46.46 (7.29)	14.70**	–		
		Class 3	65.38 (2.56)	9.57*	6.12*	–	

	Emotional function	Class 1	85.29 (1.27)	–			19.40**
		Class 2	80.80 (3.67)	1.33	–		
		Class 3	75.10 (1.94)	19.26**	1.88	–	
	Cognitive function	Class 1	86.98 (1.41)	–			12.01*
		Class 2	79.36 (4.56)	2.55	–		
		Class 3	77.76 (2.44)	10.75*	0.10	–	
	Social function	Class 1	75.00 (1.73)	–			24.49**
		Class 2	53.54 (5.29)	14.88**	–		
		Class 3	63.05 (2.72)	13.71**	2.56	–	
	Global health/quality of life	Class 1	63.15 (1.30)	–			22.73**
		Class 2	47.48 (3.59)	16.80**			
Class 3		55.72 (1.95)	10.01*	4.06*		

^a^ Equality tests of means across classes; statistics were chi‐squared; degrees of freedom in the overall test was 2.

* *p* < 0.05.

** *p* < 0.001.

After the first cycle of chemotherapy, differences in physical, role, cognitive, social functions, and global health/quality of life scores followed the pattern of “All Low” > “Low pain and high sleep disturbance” and “All Low” > “All High.” For Emotional function, differences among three latent classes followed the “All Low” > “All High.”

After four cycles of chemotherapy, differences in physical and social functions followed the pattern of “All Low” > “Low pain and high sleep disturbance” and “All Low” > “All High.” For role function and global health/quality of life scores, differences among three latent classes followed “All Low” > “Low pain and high sleep disturbance” > “All High.” As for emotional and cognitive functions, differences among the three latent classes followed the “All Low” > “All High.”

### 3.5 The transition between latent profiles

LTA identified three symptom classes from T0 to T2 (Class 1: all low; Class 2, moderate; Class 3, all high), similar to those obtained from LPA (Table S3). LTA classified more cases in Class 1 (41.7%) and Class 3 (25.2%) and fewer cases in Class 2 (33.1%) before chemotherapy compared to latent profile analysis. The number of cases in Class 1 decreased at both T1 (35.8%) and T2 (42.4%), while the number of cases in Class 2 increased at T1 (43.0%) and T2 (31.1%) compared to latent profile analysis. Meanwhile, in Class 3, the cases showed a slight decrease at T1 (21.2%) but later increased at T2 (16.5%).

The LTA model specifying a three‐group solution had a satisfactory fit (entropy = 0.869). The estimated transition probabilities from one class membership to another over time and the transition patterns are shown in Table 5. Membership in three classes remained relatively stable over time, with probabilities of 0.749 staying within their groups from T0 to T2. The transition probabilities of remaining at Class 1 (0.838 from T0 to T1 and 1.000 from T1 to T2), Class 2 (0.877 from T0 to T1 and 0.727 from T1 to T2), and Class 3 (0.676 from T0 to T1 and 1.000 from T1 to T2) had high probabilities.

**TABLE 5:** Latent class transition probabilities over time.

	Estimated probability of transition at T1		
Latent class at T0	Class 1	Class 2	Class 3
Class 1	0.838	0.153	0.009
Class 2	0.000	0.877	0.123
Class 3	0.000	0.324	0.676

	Estimated probability of transition at T2		
Latent class at T1	Class 1	Class 2	Class 3
Class 1	1.000	0.000	0.000
Class 2	0.142	0.727	0.131
Class 3	0.000	0.000	1.000

Transition patterns from T0 → T1 → T2			
Pattern	T0 → T1 → T2	Transition probabilities	
Pattern 1	Class 1 → Class 1 → Class 1	0.358	
Pattern 2	Class 1 → Class 2 → Class 1	0.020	
Pattern 3	Class 1 → Class 2 → Class 2	0.033	
Pattern 4	Class 1 → Class 3 → Class 3	0.007	
Pattern 5	Class 2 → Class 2 → Class 1	0.040	
Pattern 6	Class 2 → Class 2 → Class 2	0.232	
Pattern 7	Class 2 → Class 2 → Class 3	0.013	
Pattern 8	Class 2 → Class 3 → Class 3	0.046	
Pattern 9	Class 3 → Class 2 → Class 1	0.007	
Pattern 10	Class 3 → Class 2 → Class 2	0.046	
Pattern 11	Class 3 → Class 2 → Class 3	0.040	
Pattern 12	Class 3 → Class 3 → Class 3	0.159	

## 4 DISCUSSION

In our study, latent profile analysis identified three distinct latent classes each time based on patients' experiences of fatigue, pain, and sleep disturbance. Similar grouping results were found in a study including gynecological cancers receiving chemotherapy,^37^ which also identified three subgroups with distinct experiences within the symptom cluster of pain, fatigue, sleep disturbance, and depressive symptoms using LPA. These subgroups were labeled “low,” “moderate,” and “high.” Lee et al.^15^ used LPA to divide breast cancer patients into four subgroups based on these four symptoms. Although the subgroup results differed from ours, there was also an “overall high‐symptom burden group.” Furthermore, it's essential to acknowledge that LPA is an exploratory analysis technique, and the formation of latent classes is based on the similarity of patient responses to fatigue, pain, and sleep disturbances. As a result, differences in the number and characteristics of latent classes between studies may be attributed to disease type, sample size, and the demographic characteristics of the included research subjects.^11^

Consistent with some prior symptom cluster longitudinal studies in cancer patients undergoing chemotherapy,^14^, ^38^ the current study also found that a severe symptom burden group remained relatively stable across different treatment stages. The probabilities of staying in the “All High” class were high from T0 to T2. It indicates that specific individuals consistently experience a severe symptom burden throughout their cancer treatment journey. The “All High” group is a prioritized risk group requiring intense supportive intervention by the health care staff. Additionally, symptom experiences can change as patients progress through treatment. The transition probabilities from “Moderate” to “All High” were 0.123 from T0 to T1 and 0.131 from T1 to T2, while the probabilities of remaining in the same group were 0.877 and 0.727 from T0 to T1 and T1 to T2, respectively. It suggested that these two transition types of patients need intensive clinical monitoring and additional supportive interventions to release their symptom burden. Identifying latent class membership and the transition of latent classes over time had valuable implications for tailoring interventions to address different levels of symptom severity among cancer patients.^22^ It also allows for a nuanced understanding of the stages of symptom burden and symptom patterns at each treatment stage. Clinical staff can offer more personalized supportive care to ovarian cancer patients by anticipating the symptom category and predicting the trend of symptom changes during the chemotherapy cycle. This approach allows for a more precise and tailored approach to addressing the patient's individual needs. The persistent presence of a high‐symptom subgroup underscores the importance of ongoing monitoring and support for this specific population to improve their overall well‐being and quality of life.

Our findings revealed an association between anxiety and depression symptoms and the membership of latent classes within the symptom cluster. Moreover, depression emerged as a consistent predictor of membership in the high‐symptom class compared to the low‐symptom class throughout the chemotherapy. This association suggests that severe anxiety and depression can contribute to an increased symptom burden among ovarian cancer patients. Several previous studies^39^, ^40^ have also highlighted the relationship between anxiety, depression, and symptoms such as fatigue, pain, and sleep disturbances. Owing to the long duration of ovarian cancer, the disease burden and altered sexual function caused by anticancer treatment can cause psychological distress, leading to anxiety or depression, which can exacerbate the experience of symptoms.^41^, ^42^, ^43^ Health care professionals play a crucial role in providing comprehensive care for cancer patients. This includes strengthening psychological support, effective communication, and education to help patients better understand the disease, its treatment side effects, and the importance of maintaining confidence in their anticancer treatment. Additionally, the association between a lower educational background and membership in the high‐symptom class is consistent during chemotherapy. This aligns with previous studies^37^, ^44^ indicating that patients with lower education levels are more likely to experience a higher symptom burden. Education can empower individuals with greater health care knowledge and equip them with more effective strategies for coping with illness and symptoms.^45^ Therefore, health care providers could pay special attention to patients with lower educational backgrounds, assessing their experiences and capacity to self‐manage symptoms.

Patients in our “All High” class reported significantly lower functional status and experienced a lower quality of life. Another study comparing the quality of life between latent class membership in cancer patients had a similar finding.^46^ Our findings suggest that the classic classification of symptom clusters in ovarian cancer patients may help explain differences in quality of life. Interventions on improving quality of life may focus on symptom management, such as interventions tailored to the characteristics of different subgroups of cancer patients.

### 4.1 Clinical implications

The result of our study, which focused on Chinese ovarian cancer patients, added evidence to the increasing body of research surrounding the unique and varying symptoms experienced by cancer patients. The fact that a high‐symptom class has been identified consistently across current and prior studies highlights the severe negative impact this specific symptom cluster has on a significant number of patients undergoing chemotherapy. Patients with different symptom patterns may have varying care needs. Concentrating on the severity and nature of symptom clusters can help develop targeted interventions for ovarian cancer survivors who share similar symptom experiences. By tailoring care to specific symptom clusters, health care providers can optimize resource allocation. In other words, they can focus resources where they are most needed, improving the efficiency and effectiveness of care. For example, patients in the “All Low” group may require routine care without additional interventions, as their symptom burden is relatively low. Patients in the “All High” group, with a high‐symptom burden, would benefit from comprehensive symptom interventions to alleviate their symptoms. Patients in the “Low pain and high sleep disturbance” group might benefit more from interventions focused primarily on managing sleep disturbances. This study provides a theoretical foundation for designing intervention programs tailored to different symptom cluster patterns. Besides, because the patterns of symptoms stabilize during chemotherapy, health care providers should assess patients' symptom clusters timely before chemotherapy and take early steps to address their symptom burden. In addition, we found that patients' anxiety and depression were possible influential factors in the heterogeneity of symptom cluster experiences. This suggests a psychological characteristic in the symptom assessment process to improve accuracy. In the subsequent construction of symptom interventions, we should consider the implementation of comprehensive interventions, such as a combination of physical and psychological aspects, to alleviate the burden of this symptom cluster.

As the application of latent profile analysis and latent transition analysis in symptom clusters are still in the exploratory stage, more scholars need to be involved in the future to integrate the latent subgroups of patients with symptom interventions to help reduce symptom burden and improve the quality of life collectively.

### 4.2 Limitations

There are several limitations. This study was longitudinally designed, and participants were difficult to obtain, so only 151 subjects participated. The model indices depend highly on sample size in the latent profile analysis and latent transition analysis. There might be small power to find more than three classes even if the actual number of classes in this population is larger. We only collected the data before and after the first and the fourth cycles of chemotherapy, so patients' long‐term symptom experiences remained unknown. Our participants had similar disease characteristics. Although some interference in subgrouping was reduced, we could not identify additional predictors in various subgroups. In our study, the number of cases of positive transitions (transition from “All High” or “Moderate” group to “All Low”) or negative transitions (transition from “All Low” to “Moderate” or “All High” and transition from “Moderate” to “All High” group) was small. It prevented us from analyzing the factors responsible for the transition in subgroups of ovarian cancer patients. Future studies with larger sample sizes and more clinical features should address this limitation.

## 5 CONCLUSIONS

Our study identified a stable heterogeneous experience of the symptom clusters of fatigue, pain, and sleep disturbance in ovarian cancer patients during chemotherapy. Anxiety and depression were associated with symptom subgroups. Severe symptom burden was related to poorer quality of life. These findings could help medical staff better understand the change patterns of latent clusters in ovarian cancer patients undergoing chemotherapy and could help develop proactive care strategies for cancer patients.

## AUTHOR CONTRIBUTIONS

**Yue Feng:** Formal analysis (equal); software (equal); writing – original draft (lead). **Tangwei Lin:** Data curation (equal); investigation (equal); methodology (equal). **Xingcan Liu:** Data curation (equal); investigation (equal); methodology (equal). **Xiujing Guo:** Resources (equal); software (equal). **Jing Chen:** Conceptualization (equal); funding acquisition (equal); resources (equal); supervision (equal). **Xue Deng:** Resources (equal); supervision (equal); writing – review and editing (equal).

## FUNDING INFORMATION

This research was supported by the Basic Research Project by the Department of Nursing of West China Second University Hospital, Sichuan University (grant number HLBKJ202122).

## CONFLICT OF INTEREST STATEMENT

The authors declared that they have no conflict of interest.

## ETHICS STATEMENT

Approval was granted by the Ethics Committee of West China Second University Hospital, Sichuan University (2021‐038). Our study was conducted following the Declaration of Helsinki and in compliance with China's relevant guidelines and regulations. The participants were fully informed of the risks and benefits of the study and provided written consent.

## Supporting information


Table S1.

## Data Availability

Data could be obtained from correspondence upon reasonable request.
