# Supplementary material for: A longitudinal study of symptom cluster latent profiles in ovarian cancer patients undergoing chemotherapy
Source: Cancer Med. 2024 Mar 28;13(7):e7139. doi: 10.1002/cam4.7139 (PMC10973880; doi:10.1002/cam4.7139)
Supplement: Supplementary file 1 — Table S1. [file CAM4-13-e7139-s001.docx]

**Supplementary Table 1.** Fit indices for One- Through Five-Latent Class Solutions during chemotherapy

|  | Model | Log(L) | AIC | BIC | aBIC | Entropy | LMR-*P* | BLRT-*P* |  |
| --- | --- | --- | --- | --- | --- | --- | --- | --- | --- |
| T0 | 1-Class | -1224.228 | 2460.456 | 2478.559 | 2459.570 | n/a | n/a | n/a |  |
|  | 2-Class | -1192.738 | 2405.475 | 2435.648 | 2403.999 | 0.911 | 0.015* | <0.001** | 0.09/0.91 |
|  | **3-Class** | **-1178.291** | **2384.582** | **2426.824** | **2382.515** | **0.902** | **0.036*** | **<0.001**** | **0.08/0.33/0.59** |
|  | 4-Class | -1166.627 | 2369.255 | 2423.566 | 2366.598 | 0.887 | 0.067 | <0.001** | 0.08/0.09/0.50/0.33 |
|  | 5-Class | -1161.027 | 2366.055 | 2432.435 | 2362.807 | 0.912 | 0.775 | 0.286 | 0.01/0.22/0.03/0.04/0.70 |
| T1 | 1-Class | -1224.822 | 2461.644 | 2479.747 | 2460.758 | n/a | n/a | n/a |  |
|  | 2-Class | -1190.015 | 2400.029 | 2430.202 | 2398.553 | 0.778 | 0.009* | <0.001** | 0.60/0.40 |
|  | **3-Class** | **-1179.257** | **2386.514** | **2428.756** | **2384.447** | **0.799** | **0.008*** | **<0.001**** | **0.58/0.25/0.17** |
|  | 4-Class | -1174.204 | 2384.407 | 2438.718 | 2381.750 | 0.800 | 0.307 | 0.188 | 0.19/0.26/0.31/0.24 |
|  | 5-Class | -1173.500 | 2391.001 | 2457.381 | 2387.753 | 0.716 | 0.721 | 0.999 | 0.30/0.23/0.02/0.18/0.27 |
| T2 | 1-Class | -1217.383 | 2446.765 | 2464.869 | 2445.880 | n/a | n/a | n/a |  |
|  | 2-Class | -1190.866 | 2401.731 | 2431.904 | 2400.255 | 0.842 | 0.002* | <0.001** | 0.21/0.79 |
|  | **3-Class** | **-1180.296** | **2388.592** | **2430.834** | **2386.525** | **0.807** | **0.022*** | **<0.001**** | **0.13/0.68/0.19** |
|  | 4-Class | -1175.910 | 2387.820 | 2442.131 | 2385.163 | 0.751 | 0.656 | 0.999 | 0.15/0.20/0.33/0.32 |
|  | 5-Class | -1171.818 | 2387.635 | 2454.015 | 2384.388 | 0.791 | 0.252 | 0.250 | 0.22/0.20/0.28/0.27/0.03 |

Abbreviations: AIC, Akaike information criterion; BIC, Bayesian information criterion; aBIC, sample size adjusted BIC; BLRT, Bootstrapped Likelihood Ratio Test; LMR, Lo-Mendell-Rubin Likelihood Ratio Test. Log (L), log-likelihood;

**P*<0.05, ***P*<0.001

**Supplementary Table 2.** Distribution of demographic, clinical characteristics, and anxiety, depression symptoms between latent classes over time

| Variables | Class 1 | Class 2 | Class 3 | Statistics |
| --- | --- | --- | --- | --- |
| **T0** |  |  |  |  |
| Age, Mean±SD | 54.28±12.97 | 51.84±11.67 | 53.58±10.50 | F=0.684 |
| BMI-T0, Mean±SD | 22.14±3.53 | 21.93±2.70 | 22.64±2.87 | F=0.327 |
| Education Background, n (%) |  |  |  | χ2=3.464 |
| Junior high school and below | 20(40.0%) | 34(38.2%) | 8(66.7%) |  |
| Senior high school and upper | 30(60.0%) | 55(61.8%) | 4(33.3%) |  |
| Work Status, n (%) |  |  |  | χ2=0.644 |
| Retired/unemployed | 27(54.0) | 51(57.3) | 8(66.7) |  |
| Employed | 23(46.0) | 38(42.7) | 4(33.3) |  |
| Marriage Status, married, n (%) | 42(84.0) | 74(83.1) | 10(83.3) | χ2=0.121 |
| Stages of Cancer, n (%) |  |  |  | χ2=3.039 |
| Stage I, II | 15(30.0) | 40(44.9) | 5(41.7) |  |
| Stage III, IV | 35(70.0) | 49(55.1) | 7(58.3) |  |
| Metastasis, yes, n (%) | 18(36.0) | 19(21.3) | 4(33.3) | χ2=3.852 |
| Anxiety-T0, Median [P_25_, P_75_] | 1.0[0,3.0] | 3.0[1.0,5.0] | 3.5[1.5,5.5] | H=11.462* |
| Depression-T0, Median [P_25_, P_75_] | 1.5[0,4.0] | 3.0[1.0,4.0] | 3.0[3.0,4.0] | H=5.842 |
| **T1** |  |  |  |  |
| Age, Mean±SD | 51.99±12.95 | 54.84±7.58 | 53.29±12.22 | F=0.589 |
| BMI-T1, Mean±SD | 21.96±2.78 | 21.86±3.55 | 22.16±295 | F=0.090 |
| Education Background, n (%) |  |  |  | χ2=6.234* |
| Junior high school and below | 35(39.8) | 6(24.0) | 21(55.3) |  |
| Senior high school and upper | 53(60.2) | 19(76.0) | 17(44.7) |  |
| Work Status, n (%) |  |  |  | χ2=0.166 |
| Retired/unemployed | 49(55.7) | 15(60.0) | 22(57.9) |  |
| Employed | 39(44.3) | 10(40.0) | 16(42.1) |  |
| Marriage Status, married, n (%) | 71(80.7) | 22(88.0) | 33(86.8) | χ2=0.967 |
| Stages of Cancer, n (%) |  |  |  | χ2=2.235 |
| Stage I, II | 32(36.4) | 9(36.0) | 19(50.0) |  |
| Stage III, IV | 56(63.6) | 16(64.0) | 19(50.0) |  |
| Metastasis, yes, n (%) | 23(26.1) | 6(24.0) | 12(31.6) | χ2=0.548 |
| Anxiety-T1, Median [P_25_, P_75_] | 2.5[1.0, 4.0] | 2.0[0, 4.0] | 4.0[3.0, 5.0] | H=8.152* |
| Depression-T1, Median [P_25_, P_75_] | 2.0[1.0, 3.5] | 2.0[1.0, 4.0] | 3.5[2.0, 5.0] | H=12.705* |
| **T2** |  |  |  |  |
| Age, Mean±SD | 51.99±12.29 | 55.68±10.01 | 53.72±12.22 | F=0.866 |
| BMI-T2, Mean±SD | 22.30±3.00 | 21.77±2.83 | 21.92±3.34 | F=0.348 |
| Education Background, n (%) |  |  |  | χ2=4.160 |
| Junior high school and below | 41(39.8) | 5(26.3) | 16(55.2) |  |
| Senior high school and upper | 62(60.2) | 14(73.7) | 13(44.8) |  |
| Work Status, n (%) |  |  |  | χ2=2.132 |
| Retired/unemployed | 56(54.4) | 10(52.6) | 20(69.0) |  |
| Employed | 47(45.6) | 9(47.4) | 9(31.0) |  |
| Marriage Status, married, n (%) | 83(80.6) | 19(100) | 24(82.8) | χ2=3.862 |
| Stages of Cancer, n (%) |  |  |  | χ2=3.187 |
| Stage I, II | 36(35.0) | 9(47.4) | 15(51.7) |  |
| Stage III, IV | 67(65.0) | 10(52.6) | 14(48.3) |  |
| Metastasis, yes, n (%) | 31(30.1) | 4(21.1) | 6(20.7) | χ2=1.421 |
| Anxiety-T2, Median [P_25_, P_75_] | 3.0[1.0,4.0] | 3.0[2.0,6.0] | 4.0[3.0,6.0] | H=10.503* |
| Depression-T2, Median [P_25_, P_75_] | 2.0[1.0,4.0] | 4.0[2.0,5.0] | 4.0[2.0,6.0] | H=12.484* |

******P*<0.05, ***P*<0.001

**Supplementary Table 3.** Symptom Severity Scores Compared by Latent Classes from Latent Transition Analysis (n=151)

|  |  | Fatigue | |  | Pain | |  | Sleep disturbance | |
| --- | --- | --- | --- | --- | --- | --- | --- | --- | --- |
|  | Latent classes | Median [P_25_, P_75_] | Omnibus test *P*-value;  Post hoc contrasts |  | Median [P_25_, P_75_] | Omnibus test *P*-value;  Post hoc contrasts |  | Median [P_25_, P_75_] | Omnibus test *P*-value;  Post hoc contrasts |
| T0 | C1 (n=63) | 47.00 [43.00, 50.00] | H=20.020**  C1>C2; C1>C3 |  | 1.00 [0, 2.00] | H=27.536**  C1<C2; C1<C3 |  | 4.00 [3.00, 5.00] | H=116.985**  C1<C2<C3 |
|  | C2 (n=50) | 43.50 [39.75, 47.25] |  |  | 2.00 [1.00, 3.00] |  |  | 7.00 [6.00, 8.00] |  |
|  | C3 (n=38) | 40.00 [32.75, 44.25] |  |  | 2.00 [2.00, 4.00] |  |  | 12.00 [10.00, 13.00] |  |
| T1 | C1 (n=54) | 35.50 [31.50,42.25] | H=6.087*  C1>C3 |  | 0.50 [0, 2.00] | H=43.071**  C1<C2; C1<C3 |  | 5.00 [3.00, 5.00] | H=112.950**  C1<C2<C3 |
|  | C2 (n=65) | 34.00 [29.00, 39.00] |  |  | 2.00 [1.50, 4.00] |  |  | 8.00 [7.00, 10.00] |  |
|  | C3 (n=32) | 32.00 [26.25, 34.75] |  |  | 4.00 [2.00, 5.00] |  |  | 12.00 [11.00, 13.00] |  |
| T2 | C1 (n=64) | 38.50 [34.00, 42.75] | H=15.186**  C1>C3 |  | 1.00 [0, 2.00] | H=34.615**  C1<C2<C3 |  | 4.50 [4.00, 5.00] | H=123.770**  C1<C2<C3 |
|  | C2 (n=47) | 36.00 [30.00, 41.00] |  |  | 2.00 [1.00, 3.00] |  |  | 8.00 [7.00, 9.00] |  |
|  | C3 (n=40) | 32.00 [28.00, 38.75] |  |  | 4.00 [2.00, 5.00] |  |  | 12.00 [11.00, 13.00] |  |

**P*<0.05, ***P*<0.001;
